# Rice Quality-Related Metabolites and the Regulatory Roles of Key Metabolites in Metabolic Pathways of High-Quality Semi-Glutinous *japonica* Rice Varieties

**DOI:** 10.3390/foods11223676

**Published:** 2022-11-17

**Authors:** Jinyan Zhu, Ao Li, Changhui Sun, Jiao Zhang, Jinlong Hu, Shuai Wang, Nianbing Zhou, Qiangqiang Xiong

**Affiliations:** 1 Jiangsu Key Laboratory of Crop Genetics and Physiology/Jiangsu Key Laboratory of Crop Cultivation and Physiology, Agricultural College of Yangzhou University, Yangzhou 225009, China; 2 Jiangsu Co-Innovation Center for Modern Production Technology of Grain Crops, Yangzhou University, Yangzhou 225009, China

**Keywords:** semi-glutinous *japonica* rice, metabolomics, rice quality, eating quality, metabolic pathway

## Abstract

We explored the related metabolites produced by different quality semi-glutinous *japonica* rice varieties and the modulatory role of key metabolites in metabolic mechanisms. In this study, three high-quality edible semi-glutinous rice varieties were employed as investigational materials, the metabolites of the three varieties were detected using LC–MS metabolomics technology, and the rice quality traits of the three rice varieties were determined. The taste value (TV) of Yangnongxiang 28 (YNX28H) was substantially higher than that of Hongyang 5 hao (HY5H) and Nanjing 5718 (NJ5718), and the hardness (HA) of YNX28H was significantly lower than that of HY5H and NJ5718. The HA was significantly negatively correlated with the TV. The highest chalkiness rate (CR) and chalkiness degree (CD) were observed for NJ5718, and the lowest CR and CD were observed for HY5H. HY5H had a substantially lower protein content (PC) than YNX28H and NJ5718 and a markedly higher amylose content (AC) than those two varieties. Overall, 188 differential metabolites (DMs) were recognized between HY5H and NJ5718. A total of 136 DMs were detected between YNX28H and NJ5718, and 198 DMs were recognized between HY5H and YNX28H. The metabolites with a strong correlation with rice quality were mainly associated with amino acid metabolism, lipid metabolism and the citrate cycle. The key metabolites in the metabolic pathway include lipid metabolites (sagittariol, glycerophosphocholine, gamma-eudesmol rhamnoside, goshonoside F1, diosbulbinoside F, and corchorifatty acid F), amino acid metabolites (pantothenic acid, L-serine, L-proline, L-aspartic acid, L-glutamate, L-asparagine, and glutathione) and carbohydrate metabolites (sucrose, levan, D-maltose, and amylose). These key metabolites play important regulatory roles in metabolic mechanisms, providing a theoretical basis for breeding new high-quality edible rice varieties.

## 1. Introduction

With the progress achieved in the social economy and the increasing global reach of the international rice market, both consumers and the market have demanded higher requirements for rice production. Semi-glutinous *japonica* rice varieties are intermediate varieties between ordinary *japonica* rice and glutinous rice and are characterized by their endosperm amylose content (AC) [1]. Semi-glutinous rice has a glossy and translucent surface, soft and elastic texture, resistance to hardening at cold temperatures, and an excellent eating quality [2]. Semi-glutinous rice is very popular among people in Jiangsu, Zhejiang, Anhui and Shanghai, and it is also of commercial interest as a raw grain-to-rice processing enterprise and grain department. The annual cultivation area of semi-glutinous rice varieties is approximately 1 million hm^2^, accounting for half of the *japonica* rice planting area in South China, and it is still expanding [3,4]. The cultivation of high-quality semi-glutinous rice varieties has substantially improved the enthusiasm of farmers for planting and has promoted the dual development of the high-quality rice industry and seed industry. It has also made important contributions to the adjustment of the supply-side structure of the southern *japonica* rice industry and the enhancement of rice value and harvest efficiency [5].

Scientific recognition and examination of the nutritional quality of agricultural goods has a vital function in increasing the dietary level of high-quality agricultural products. Due to the complex composition of nutrients in agricultural products, existing analytical methods only analyze the concentrations and functions of known nutrients [6,7]. At present, the analysis and identification of a large number of other functional active substances in agricultural products are impossible. Metabolomics technology uses high-throughput chemical analysis technology to qualitatively and quantitatively measure small-molecule metabolites in biological samples, providing data to analyze the roles of key metabolites in metabolic pathways [8,9]. The types, content variations, and metabolic production of various nutrients in crops have all been well examined [10,11,12], and databases of dietary quality metabolism of rice and other main products have been established. Metabolites in rice grains might be used to assess the types and compositions of metabolites with significant effects on rice quality and to completely represent the entire metabolic state of rice seeds [13,14,15]. Using metabolomic techniques, we explored changes in the quantity, species and abundance of metabolites as a function of nitrogen fertilization and planting concentration and their ultimate effects on rice quality [16]. The differences in the processing quality, AC, and taste value (TV) under altered nitrogen fertilizer and planting density treatments and their connection with metabolites were analyzed [16]. Among them, lipid metabolites are a significant component influencing the TV of rice [16]. In addition, the important metabolites of purple glutinous rice were analyzed using metabolomics technology, and six metabolites were identified as candidate metabolite biomarkers for assessing the glutinous rice antioxidant capacity [17]. The regulatory functions of important metabolites in metabolic processes were then defined after an additional analysis of the metabolites related to the nutrients of purple glutinous rice [18].

In this study, with the advantage of metabolomics, differences in quality and changes in the quantity, the species and abundance of metabolites in three semi-glutinous *japonica* rice varieties were studied. The TVs of the three rice varieties differ, but rice varieties of the same type are more conducive to exploring the relationship between metabolites and TVs. This study further explored the metabolites related to the formation of semi-glutinous *japonica* rice quality, and the functions of vital metabolites in metabolic mechanisms were analyzed, providing basic data for metabolomics-assisted breeding and metabolic engineering strategies.

## 2. Materials and Methods

### 2.1. Materials and Growth Conditions of Plants

The investigated materials were three semi-glutinous conventional *japonica* rice varieties: Hongyang 5 hao (HY5H), Yangnongxiang 28 (YNX28H), and Nanjing 5718 (NJ5718). The three rice varieties were planted in the Shatou base of Yangzhou University. The rice crop was sown on 22 May 2021. The raised blanket seedlings were 25 days old when they were first planted. Three leaves were present when the rice seedlings were transplanted at one stage. Four seedlings were planted in each hole, and each variety was planted in triplicate. The rice was planted in high standard farmland with good drainage and irrigation conditions. Compound fertilizer was used for rice fertilization, with the proportions of nitrogen, phosphorus and potassium accounting for 15%. A total of 270 kg hm^−2^ of pure nitrogen was applied. The proportions of the base, tiller, and panicle fertilizers were 5:3:2. In accordance with the needs of traditional high-yield rice cultivation, diseases, pests, and weeds were managed.

### 2.2. Detection of Rice Quality Traits

The rice was harvested at the mature stage. Three biological replicates of each rice variety were collected, threshed, naturally dried, and stored for three months. The rice seeds has a water content of approximately 14%. The national standard of the People’s Republic of China (GB/T17891-2017) was used to determine the brown rice rate (BR), milled rice rate (MR), head milled rice rate (HMR), chalkiness rate (CR), chalkiness degree (CD), gel consistency (GC), protein content (PC), and AC. A rice taste meter (STA 1A, Satake, Japan) was used to test the hardness (HA), appearance (AP), balance degree (BD), and viscosity (VI) of rice varieties, and the complete results were computed (TV).

### 2.3. Sample Metabolite Obtaining

We used the Xiong et al. (2022 a, b) technique and used precise weights to measure the samples of head milled rice (50 mg) [16,17] to extract metabolites. The 50 mg head-milled rice sample was accurately weighed into a 2 mL centrifuge tube, and a 6 mm diameter bead was added. Then, 400 µL of an extract (methanol:water = 4:1 (*v*:*v*)) containing 0.02 mg mL^−1^ internal standard (L-2-chlorophenylalanine) was added to the centrifuge tube. The sample solution was first extracted using reduced-temperature ultrasonic extraction for 30 min (5 °C, 40 kHz) after being ground for 6 min in a frozen tissue grinder at −10 °C and 50 Hz. After an incubation at −20 °C for 30 min, the samples were centrifuged for 15 min (13,000× *g*, 4 °C), and the supernatant was then transferred to a sample vial with an internal cannula for analysis. Additionally, 20 µL of the supernatant from each sample was pipetted together as a quality control (QC) sample.

### 2.4. LC–MS/MS Analysis

Chromatographic separation of the metabolites was conducted via a Thermo UHPLC system with an ACQUITY UPLC HSS T3 column (100 mm × 2.1 mm i.d., 1.8 µm; Waters, Milford, CT, USA). The following chromatographic conditions were used: the injection volume was 2 μL, the column temperature was 40 °C, the mobile phase A composition was 95.5% water and 5% acetonitrile (including 0.1% formic acid), and the mobile phase B composition was 47.5% acetonitrile, 47.5% isopropanol, and 5% water (including 0.1% formic acid). The mass spectrometric data were collected using a Thermo UHPLC-Q Exactive HF-X mass spectrometer with an ESI source running in either positive or negative ion mode. The ideal conditions were as follows: normalized collision energy, 20–40–60 V rolling for MS/MS; heater temperature, 425 °C; capillary temperature, 325 °C; sheath gas flow rate, 50 arb; aux gas flow rate, 13 arb; and ion-spray voltage floating (ISVF), −3500 V in negative mode and 3500 V in positive mode. The resolution of the MS/MS scan was 7500, while the resolution of the entire MS scan was 60,000. The data-dependent acquisition mode was used to record the data. A mass range of 70–1050 m/z was employed for identification.

### 2.5. Differential Metabolite Analysis

On the basis of variable importance, in projection values greater than 1 and *p* values less than 0.05, differential metabolites (DMs) were selected. Through the use of the Majorbio Cloud Platform (https://cloud.majorbio.com, accessed on 1 July 2022), a multivariate statistical evaluation was conducted with the ropls (version 1.6.2, http://bioconductor.org/packages/release/bioc/html/ropls.html, accessed on 1 July 2022) R package from Bioconductor. After metabolic enrichment and pathway analysis using a database search (Kyoto Encyclopedia of Genes and Genomes (KEGG), http://www.genome.jp/kegg/, accessed on 1 July 2022), the DMs were mapped to their respective biochemical mechanisms. These metabolites may be divided into groups based on the processes in which they participate or the functions that they perform. Enrichment analysis typically examines the node to ascertain if a collection of metabolites is present in a function node. The idea is that an annotated study of one metabolite would eventually lead to an examination of several metabolites. Through the use of Fisher’s exact test, the Python module scipy.stats (https://docs.scipy.org/doc/scipy/, accessed on 1 July 2022) was employed to identify statistically significantly enriched mechanisms.

### 2.6. Data Analysis

The WPS 2021 program was employed to sort the relevant information and estimate the average value. SPSS 18.0 software was employed to analyze the variance in rice quality data, and Adobe Illustrator CS6 software was utilized to prepare various figures.

## 3. Results

### 3.1. Rice Quality Traits of Three Semi-Glutinous japonica Rice Varieties

The alterations in rice quality traits among the three semi-glutinous *japonica* rice varieties were analyzed (Table 1). The BR of YNX28H was 0.74% and 1.09% greater than those of HY5H and NJ5718, respectively. The BR of HY5H was 3.52% and 3.13% greater than those of YNX28H and NJ5718, respectively. The lowest HMR was observed for HY5H, which was significantly different from the values of YNX28H and NJ5718. The highest CR and CD were observed for NJ5718, while those of HY5H were the lowest. The GC of YNX28H was 3.84% and 12.04% higher than those of HY5H and NJ5718, respectively. The AP of YNX28H was 20.87% and 17.11% greater than those of HY5H and NJ5718, respectively. The HA of YNX28H was significantly less than those of HY5H and NJ5718. The VI values of the three semi-glutinous *japonica* rice varieties YNX28H, HY5H and NJ5718 were not significantly different. The BD of YNX28H was 12.97% and 17.54% higher than those of HY5H and NJ5718, respectively. The TV of YNX28H was 7.89% and 9.69% higher than those of HY5H and NJ5718, respectively. The AC of HY5H was significantly higher than those of YNX28H and NJ5718, and the PC of HY5H was substantially lower than those of YNX28H and NJ5718. Correlation analysis of the rice quality traits was performed (Table S1). HMR, CD, and CR were substantially negatively correlated with AC. AP and VI were markedly positively correlated with BD and TV. HA was considerably negatively correlated with BD and TV. AC was substantially negatively correlated with PC.

### 3.2. Multivariate Statistical Analysis

**Figure 1:**
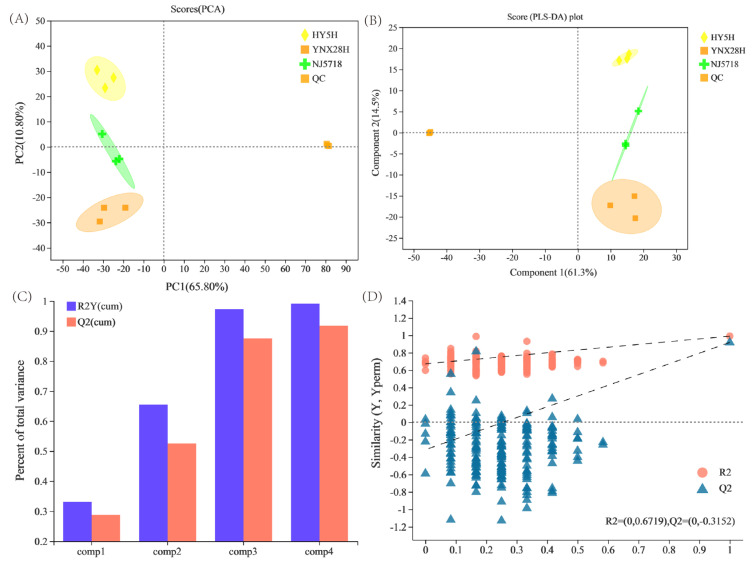
Testing for permutations and multivariate statistical scores among the three *japonica* rice varieties. (**A**) PCA, (**B**) PLS-DA, (**C**) overview of the PLS-DA model, and (**D**) PLS-DA permutation testing. PCA, principal component analysis; PC, principal component; PLS-DA, partial least squares discriminant analysis; HY5H, Hongyang 5 hao; YNX28H, Yangnongxiang 28; NJ5718, Nanjing 5718.

**Figure 2:**
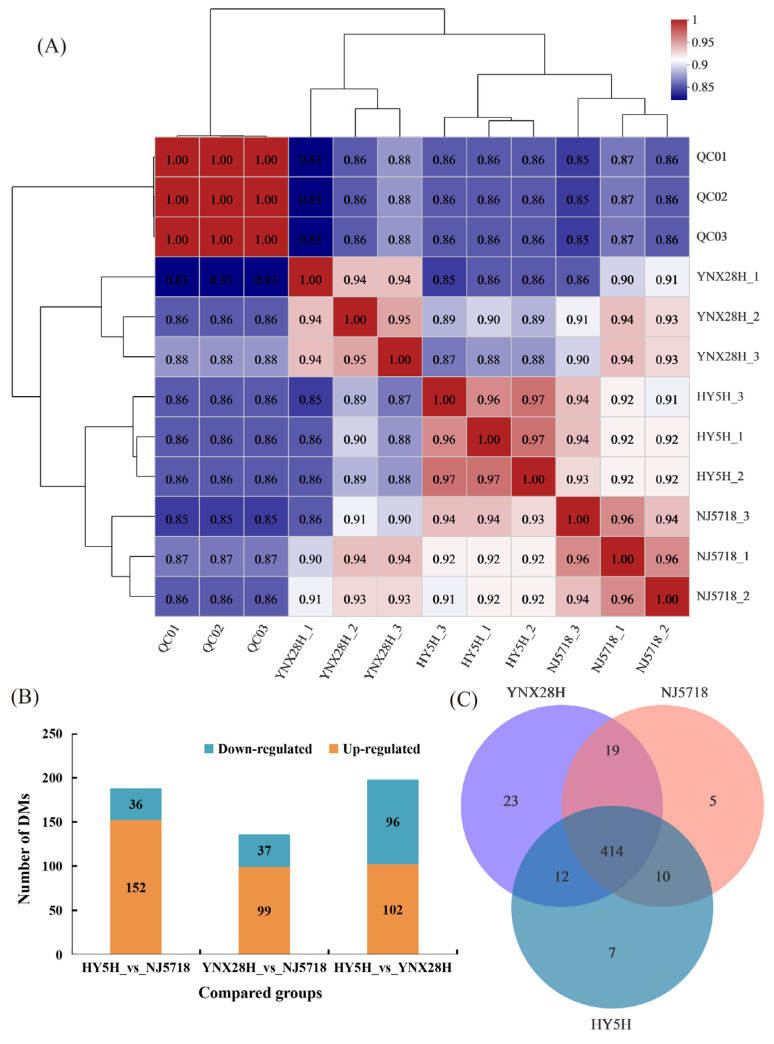
Sample reproducibility and metabolite analysis. (**A**) Correlation heatmap of three semi-glutinous *japonica* rice grain samples. The right and inferior sides in the figure are the sample names. (**B**) Pairwise comparison of DMs. (**C**) Venn distribution of metabolites. HY5H, Hongyang 5 hao; YNX28H, Yangnongxiang 28; NJ5718, Nanjing 5718.

First, metabolite data from three types of semi-glutinous *japonica* rice were subjected to principal component analysis (PCA). The first two principal components (PCs), PC1 and PC2, on the PCA score table were responsible for 65.8% and 10.8%, respectively, of the data variance (Figure 1A). Components 1 and 2 were shown to explain 61.3% and 14.5% of the variation, respectively, via partial least squares discriminant analysis (PLS-DA) (Figure 1B). The explanatory capability and predictive capability of the PLS-DA model were assessed using R2Y and Q2, respectively. The model was more stable and reliable for greater cumulative R2Y and Q2 values. Together, the cumulative R2Y value and the cumulative Q2 value were 0.987 and 0.821, respectively (Figure 1C). After 200 replacement tests, the R2 and Q2 values, estimated by random arrangement in any replacement examination, were smaller than the original values, and the slope of the straight line was large (Figure 1D). PLS-DA did not display model overfitting, and the DMs were screened according to the VIP value. The correlation coefficient between samples was higher, and the lowest correlation coefficient between samples was 0.94, showing that the repeatability between samples was sufficient (Figure 2A).

### 3.3. Metabolic Profiling

**Figure 3:**
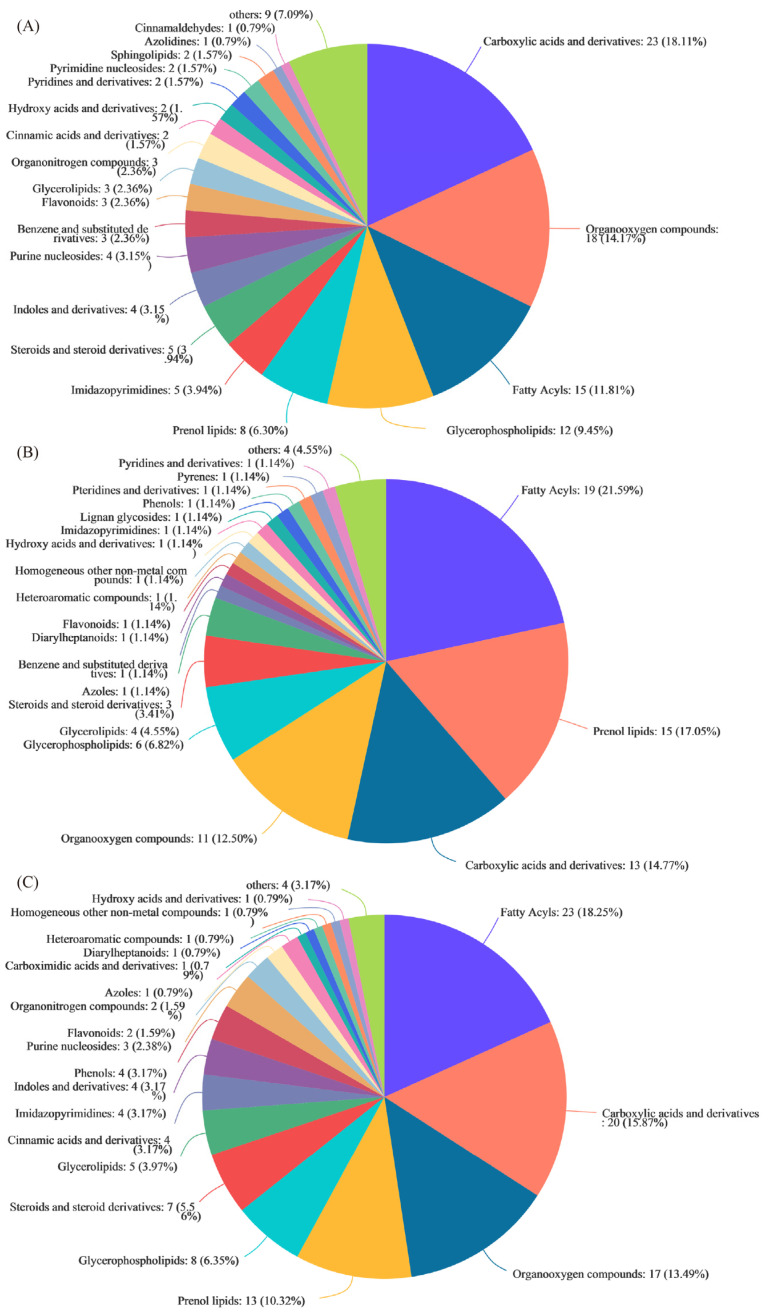
Statistical map of compounds. (**A**) HY5H_vs_NJ5718, (**B**) YNX28H_vs_NJ5718, and (**C**) HY5H_vs_YNX28H. HY5H, Hongyang 5 hao; YNX28H, Yangnongxiang 28; NJ5718, Nanjing 5718.

A total of 188 DMs were identified between HY5H and NJ5718 (152 upregulated and 36 downregulated) (Figure 2B; Table S2). Glycerophospholipids accounted for 9.45% of the detected DMs. Prenol lipids formed 6.30% of the total DMs. Flavonoids comprised 2.36% of the total DMs. Glycerolipids accounted for 2.36%, phenols represented 0.79%, and coumarins and their derivatives accounted for 0.79% of the DMs (Figure 3A). A total of 136 DMs were identified between YNX28H and NJ5718 (99 upregulated and 37 downregulated) (Figure 2B; Table S3). Prenol lipids accounted for 17.05% of the detected DMs. Glycerophospholipids accounted for 6.82%, glycerolipids accounted for 4.55%, lignan glycosides accounted for 1.14%, phenols accounted for 1.14%, and flavonoids accounted for 1.14% of DMs (Figure 3B). A total of 198 DMs were identified between HY5H and YNX28H (102 upregulated and 96 downregulated) (Figure 2B; Table S4). Prenol lipids accounted for 10.32% of the detected DMs. Glycerophospholipids represented 6.35%, glycerolipids accounted for 3.97%, cinnamic acids and their derivatives accounted for 3.17%, phenols accounted for 3.17%, flavonoids accounted for 1.59%, and lignan glycosides comprised 0.79% of the DMs (Figure 3C). The three varieties had 414 common metabolites, and each variety also had its own unique metabolites (Figure 2C). In addition, we visualized the metabolites of each comparison group in a volcano map (Figure S1A–C).

### 3.4. KEGG Pathways

Plant metabolism often forms complex pathways and networks through different molecules, which eventually leads to systematic changes in metabonomics. Through the use of the KEGG pathway database (http://www.kegg.jp/kegg/pathway.html, accessed on 1 July 2022), we found that the DMs identified between the HY5H and NJ5718 varieties were involved in flavone and flavonol biosynthesis; pentose and glucuronate interconversion; fatty acid biosynthesis; glucosinolate biosynthesis; glycerolipid metabolism, starch and sucrose metabolism; and alpha-linolenic acid metabolism (Table S5). The DMs identified between the YNX28H and NJ5718 varieties participate in linoleic acid metabolism, the pentose phosphate pathway, glycerolipid metabolism, alpha-linolenic acid metabolism, glyoxylate and dicarboxylate metabolism, pentose and glucuronate interconversion, and starch and sucrose metabolism (Table S5). The DMs identified between the HY5H and YNX28H varieties participate in pantothenate and CoA biosynthesis, the pentose phosphate pathway, phenylpropanoid biosynthesis, pentose and glucuronate interconversions, glycerolipid metabolism, alpha-linolenic acid metabolism, starch and sucrose metabolism, and glycerophospholipid metabolism (Table S5).

### 3.5. Correlation Analysis between Rice Quality Traits and Metabolite Levels

**Figure 4:**
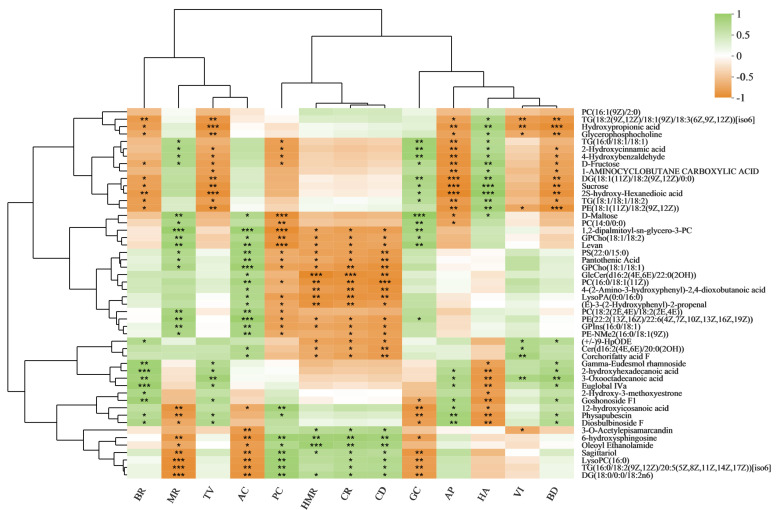
Correlation analysis of rice quality traits and DMs. * *p* < 0.05, ** *p* < 0.01, and *** *p* < 0.001. BR, brown rice rate; MR, milled rice rate; HMR, head milled rice rate; CR, chalkiness rate; CD, chalkiness degree; GC, gel consistency; PC, protein content; AC, amylose content; AP, appearance; HA, hardness; VI, viscosity; BD, balance degree; TV, taste value.

**Figure 5:**
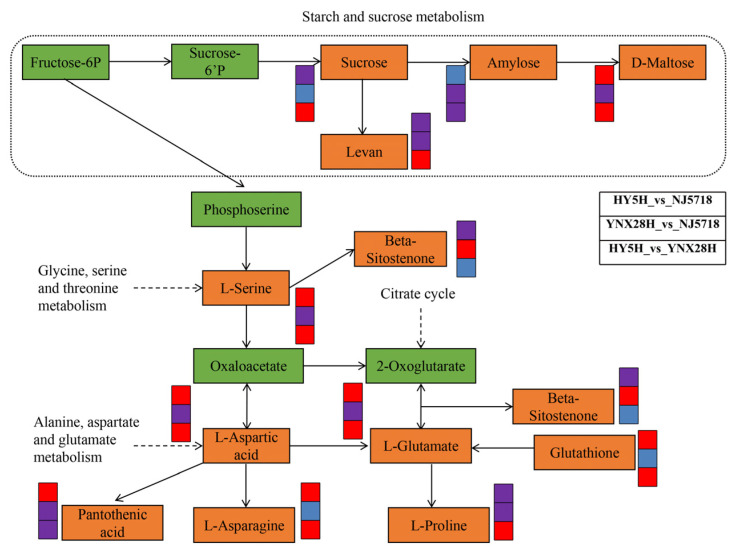
A description of a few important metabolites involved in metabolic processes. Orange rectangles display the important metabolites. Red represents upregulation, blue represents downregulation, and purple represents no significant difference. HY5H, Hongyang 5 hao; YNX28H, Yangnongxiang 28; NJ5718, Nanjing 5718.

We performed correlation analysis between rice quality traits and metabolite levels to further clarify their relationships (Figure 4). The hydroxypropionic acid and glycerophosphocholine levels were markedly negatively correlated with BR, TV, AP, VI, and BD. The hydroxypropionic acid, glycerophosphocholine, D-fructose, sucrose, and D-maltose levels were substantially positively correlated with HA. The 2-Hydroxycinnamic acid, 4-hydroxybenzaldehyde, D-fructose, and sucrose levels were considerably negatively correlated with AP, BD, and TV. The 2-Hydroxycinnamic acid, 4-hydroxybenzaldehyde, D-fructose and sucrose levels were considerably positively correlated with GC and HA. Levan and pantothenic acid levels were substantially positively correlated with MR and AC. Levan, corchorifatty acid F, and pantothenic acid levels were substantially negatively correlated with HMR, CR, and CD. Levan, pantothenic acid, 2-hydroxycinnamic acid, 4-hydroxybenzaldehyde, D-fructose, and D-maltose levels all exhibited significant negative correlations with PC. Significant positive correlations were observed between D-maltose, levan, pantothenic acid, and corchorifatty acid F levels and AC. Significant positive correlations were identified between BR, BD, and TV and gamma-eudesmol rhamnoside, 2-hydroxyhexadecanoic acid, euglobal IVa, goshonoside F1, and diosbulbinoside F levels. The sagittariol and oleoyl ethanolamide levels displayed substantial positive correlations with HMR, CR, CD, and PC. The sagittariol and oleyl ethanolamide levels were substantially negatively correlated with MR and AC. In addition, we mapped the metabolic pathways that regulate some key metabolites (Figure 5). Sagittariol, glycerophosphocholine, gamma-eudesmol rhamnoside, goshonoside F1, diosbulbinoside F, and corchorifatty acid F are lipid metabolites (Table S6). Pantothenic acid, L-serine, L-proline, L-aspartic acid, L-glutamate, L-asparagine, and glutathione are amino acid metabolites (Table S6). Sucrose, levan, D-maltose, and amylose are carbohydrate metabolites (Table S6).

## 4. Discussion

Processing, appearance, nutritional value, and eating quality are among the important factors affecting rice quality. Appearance quality is an important commodity quality of rice, while eating quality is the core index of high-quality rice [19]. The appearance quality of rice mainly includes indicators such as chalkiness [19]. In this study, the differences in quality traits among three semi-glutinous *japonica* rice varieties were analyzed (Table 1). The CR and CD of YNX28H ranked in the middle among the three semi-glutinous *japonica* varieties (Table 1). However, YNX28H had the highest TV, followed by HY5H (Table 1). The TV was significantly negatively correlated with HA (Table S1). On one hand, the eating quality of YNX28H was the best among the three semiglutinous *japonica* varieties; on the other hand, the hardness of the rice exerted a strong effect on the eating quality of the rice. The amylose content of the southern semi-glutinous *japonica* rice was lower than that of the northern *japonica* rice, which reduced the hardness of the rice to a large extent, increased the viscosity of the rice, and ultimately made the rice soft and sticky with a better texture [20]. We also observed from the variations in CR and CD that the CR and CD values of the semi-glutinous *japonica* varieties have little effect on rice eating quality (Table 1). This result is due to the cloudy appearance of the endosperm of the semi-glutinous *japonica* varieties, resulting in higher CR and CD values. More than 70% of rice is starch, which is composed of amylose and amylopectin, and the proportion of amylose directly affects the optimal cooking time, water absorption, viscosity and other indicators of rice [21,22,23], thus affecting its eating quality [20]. Previous studies have suggested that excessive amylose and protein contents are not conducive to the development of rice with a high eating quality [20]. In this study, the correlations between TV and AC and PC were not significant. We further analyzed the factors responsible for this observation in order to determine whether it occurred because the three rice varieties were all semi-glutinous *japonica* varieties, and the effects of AC and PC on TV were determined to be relatively small.

The most important metrics for evaluating the quality of a rice variety are its flavor and nutritional content. These metrics also provide appropriate experimental parameters for examining the processes that give food its flavor and nutritional value. However, few studies have examined the nutritional quality of milled rice [16]. In this study, three semi-glutinous varieties of whole milled rice were used as test materials, among which the comparison of YNX28H and NJ5718 had the lowest content of DMs (Figure 2B). Further studies are needed to determine whether this observation is related to the similar ACs of YNX28H and NJ5718. As amino acids are the building blocks of protein, they are essential nutrients for both humans and animals. Amino acids are present in cereal grains. In this study, amino acid metabolism pathways such as arginine biosynthesis and arginine and proline metabolism were enriched (Table S5). Pantothenic acid, L-serine, L-proline, L-aspartic acid, L-glutamate, L-asparagine, and glutathione are the key metabolites of amino acids (Table S6; Figure 5) that play important roles in amino acid metabolism pathways. The citrate cycle is considered the final metabolic pathway of the three major nutrients (carbohydrates, lipids, and amino acids) and is presumed to be the hub of the metabolic connection and transformation of carbohydrates, lipids, amino acids, and nucleic acids [24,25]. The contents of sucrose, levan, D-maltose, and amylose were significantly different and showed differences in enrichment in the starch and sucrose metabolism pathways in the different comparison groups (Figure 5; Table S6). This result also shows that each variety formed independently, with its own carbohydrate synthesis pathway, but a certain correlation exists, and the same key metabolites are involved in the carbohydrate synthesis pathway (Figure 5). Lipids are significant rice ingredients that affect eating quality [16]. This investigation identified several lipid metabolites related to the formation of rice quality traits and key metabolites in the metabolic pathway. Sagittariol, glycerophosphocholine, gamma-eudesmol rhamnoside, goshonoside F1, diosbulbinoside F, and corchorifatty acid F were all identified as lipid metabolites (Figure 5) with important functions in the formation of rice quality that ensured the excellent eating features of semi-glutinous *japonica* rice.

## 5. Conclusions

In this investigation, certain alterations in rice quality traits were observed among the three semi-glutinous *japonica* varieties. The MR of HY5H was 3.52% and 3.13% higher than those of YNX28H and NJ5718, respectively. The TV of YNX28H was 7.89% and 9.69% higher than those of HY5H and NJ5718, respectively. TV was significantly negatively correlated with HA. The hardness of rice strongly affects the TV. The AC of HY5H was significantly higher than those of YNX28H and NJ5718. Most metabolites were enriched in the amino acid metabolism pathway, lipid metabolism mechanism, and citrate cycle. The key metabolites in the metabolic pathway are lipid metabolites (sagittariol, glycerophosphocholine, gamma-eudesmol rhamnoside, goshonoside F1, diosbulbinoside F, and corchorifatty acid F), amino acid metabolites (pantothenic acid, L-serine, L-proline, L-aspartic acid, L-glutamate, L-asparagine, and glutathione), and carbohydrate metabolites (sucrose, levan, D-maltose, and amylose). These metabolites play important regulatory roles in metabolic pathways. Metabonomics is a new and very important technology used to study secondary metabolites. In the future, metabonomics, genomics, proteomics and other omics technologies may be combined to develop the value of secondary metabolites and secondary metabolic pathways in biological, agricultural, medical and other fields.

## Figures and Tables

**Table 1:** Analysis of differences in the quality traits of three semi-glutinous *japonica* rice varieties.

Varieties	BR	MR	HMR	CR	CD	GC	AP	HA	VI	BD	TV	AC	PC
HY5H	84.88% b	71.43% a	43.49% c	22.07 c	5.97 c	86.67 a	6.23 b	6.67 a	6.23 a	6.17 b	67.17 b	13.78% a	7.93% b
YNX28H	85.51% a	69.00% b	50.56% b	38.77 b	13.73 b	64.00 b	7.53 a	6.07 b	6.47 a	6.97 a	72.47 a	11.50% b	8.47% a
NJ5718	84.59% b	69.26% b	60.81% a	57.32 a	23.18 a	70.33 b	6.43 b	6.67 a	5.70 a	5.93 b	66.07 b	10.74% b	8.47% a

Note: The lowercase letters after the data in the same column indicate a significant difference with a *p* value at the 0.05 level.

## Data Availability

The datasets generated for this study are available on request to the corresponding author.

## Supplementary Materials

The supporting data listed below can be obtained at https://www.mdpi.com/article/10.3390/foods11223676/s1: Figure S1: DM volcano map. (A) HY5H_vs_NJ5718, (B) YNX28H_vs_NJ5718, and (C) HY5H_vs_YNX28H. Table S1: Correlation analysis of rice quality traits. Table S2: Data for DMs identified between HY5H and NJ5718. Table S3: Data for DMs identified between YNX28H and NJ5718. Table S4: Data for DMs identified between HY5H and YNX28H. Table S5: KEGG pathways associated with DMs. Table S6: Metabolite information related to rice quality traits and metabolic pathways.
