# Supplementary material for: Rice Quality-Related Metabolites and the Regulatory Roles of Key Metabolites in Metabolic Pathways of High-Quality Semi-Glutinous japonica Rice Varieties
Source: Foods. 2022 Nov 17;11(22):3676. doi: 10.3390/foods11223676 (PMC9689214; doi:10.3390/foods11223676)
Supplement: Supplementary file 1 [file foods-11-03676-s001.zip › Figure S1.pdf]

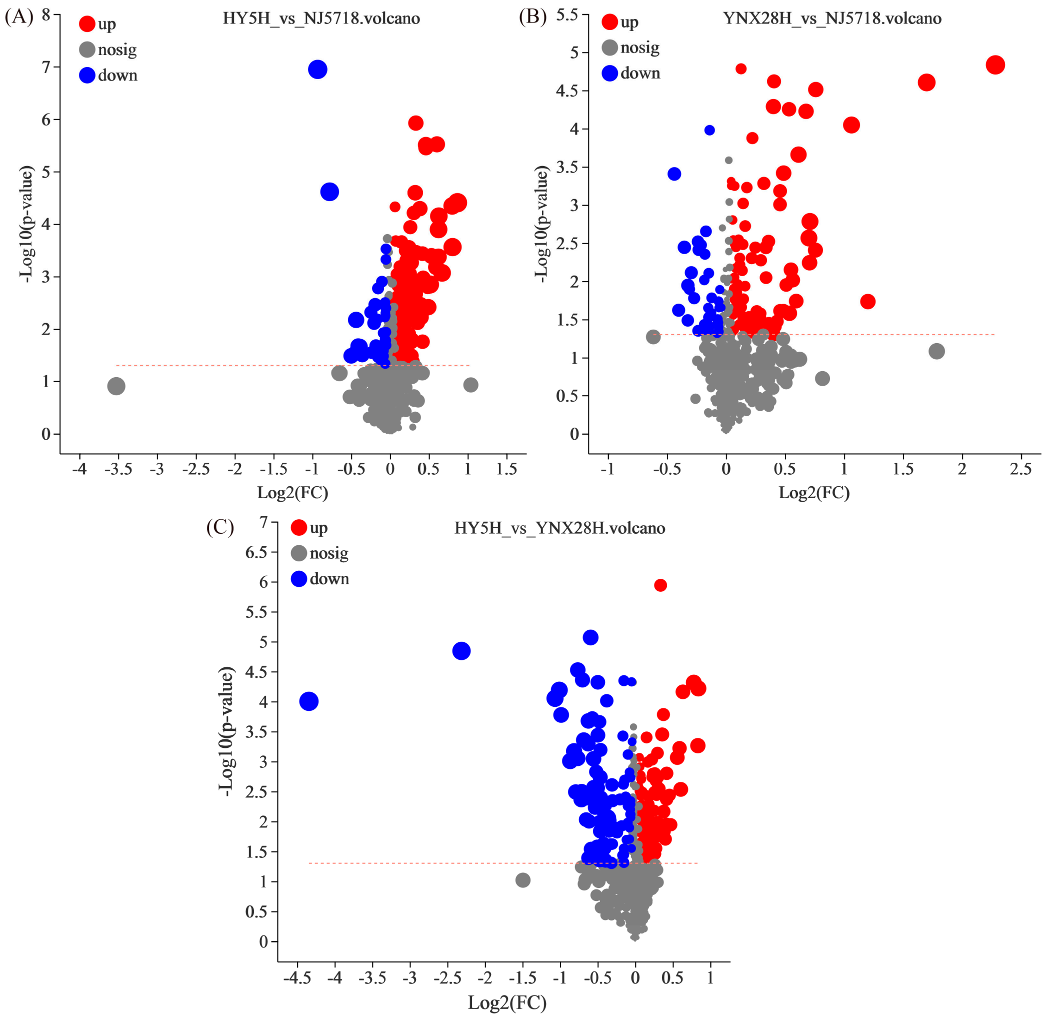

Figure S1. DM volcano map. Each dot represents a specific metabolite, and the size of the dot represents the VIP value. On the left are differentially down-regulated metabolites, and on the right are differentially up-regulated metabolites; the more left and right are, the more significant the point above. (A) HY5H_vs_NJ5718; (B) YNX28H_vs_NJ5718; (C) HY5H_vs_YNX28H.
