# Supplementary material for: Rice Quality-Related Metabolites and the Regulatory Roles of Key Metabolites in Metabolic Pathways of High-Quality Semi-Glutinous japonica Rice Varieties
Source: Foods. 2022 Nov 17;11(22):3676. doi: 10.3390/foods11223676 (PMC9689214; doi:10.3390/foods11223676)
Supplement: Supplementary file 1 [file foods-11-03676-s001.zip › Table S1.pdf]

Table S1. Correlation analysis of rice quality traits

Character	BR	MR	HMR	CR	CD	GC	AP	HA	VI	BD	TV	AC	PC
BR		-0.266	-0.364	-0.269	-0.329	-0.405	0.799**	-0.909**	0.65	0.833**	0.841**	0.051	0.213
MR			-0.698*	-0.761*	-0.767*	0.887**	-0.639	0.56	0.071	-0.344	-0.39	0.920**	-0.950**
HMR				0.962**	0.956**	-0.529	0.079	0.069	-0.37	-0.176	-0.14	-0.855**	0.752*
CR					0.991**	-0.639	0.134	0.005	-0.418	-0.165	-0.119	-0.895**	0.842**
CD						-0.622	0.087	0.049	-0.487	-0.222	-0.176	-0.915**	0.822**
GC							-0.717*	0.622	-0.021	-0.439	-0.479	0.786*	-0.919**
AP								-0.962**	0.672*	0.933**	0.950**	-0.312	0.612
HA									-0.666	-0.928**	-0.942**	0.223	-0.494
VI										0.884**	0.863**	0.4	-0.072
BD											0.998**	0.019	0.313
TV												-0.029	0.359
AC													-0.884**
PC													
