# Supplementary material for: Rice Quality-Related Metabolites and the Regulatory Roles of Key Metabolites in Metabolic Pathways of High-Quality Semi-Glutinous japonica Rice Varieties
Source: Foods. 2022 Nov 17;11(22):3676. doi: 10.3390/foods11223676 (PMC9689214; doi:10.3390/foods11223676)
Supplement: Supplementary file 1 [file foods-11-03676-s001.zip › Table S5.pdf]

Table S5. KEGG pathways associated with DMs.

Pathway Description	Pathway_ID	Ratio_in_pop	Metabolites	*P*-value
**KEGG pathways between HY5H_vs_NJ5718**				
Phenylalanine, tyrosine and tryptophan biosynthesis	map00400	34/4834	C00082	0.2886
Flavone and flavonol biosynthesis	map00944	51/4834	C04858	0.4005
Phenylalanine metabolism	map00360	60/4834	C00082	0.4525
Pentose and glucuronate interconversions	map00040	58/4834	C00474	0.4414
Fatty acid biosynthesis	map00061	58/4834	C06424	0.4414
Phenylpropanoid biosynthesis	map00940	68/4834	C00082	0.4951
Glucosinolate biosynthesis	map00966	77/4834	C00082	0.5391
Arginine biosynthesis	map00220	23/4834	C00025; C00049	0.0214
beta-Alanine metabolism	map00410	32/4834	C00049; C00864	0.0396
Pantothenate and CoA biosynthesis	map00770	30/4834	C00049; C00864	0.0352
Glyoxylate and dicarboxylate metabolism	map00630	62/4834	C00025; C00065	0.1256
Glycerolipid metabolism	map00561	38/4834	C00416	0.3166
Alanine, aspartate and glutamate metabolism	map00250	28/4834	C00025; C00049; C00152	0.0025
Glycine, serine and threonine metabolism	map00260	50/4834	C02737; C00049; C00065	0.013
Arginine and proline metabolism	map00330	78/4834	C00025; C05945; C10497	0.0416
Tryptophan metabolism	map00380	83/4834	C05645; C05835; C00643	0.0486
Starch and sucrose metabolism	map00500	37/4834	C06215; C00760;	0.0004

			C00208; C00718	
Cysteine and methionine metabolism	map00270	64/4834	C00049; C00170; C00065; C00051	0.0035
alpha-Linolenic acid metabolism	map00592	44/4834	C01226; C00157; C16316	0.0092
Arachidonic acid metabolism	map00590	75/4834	C03577; C14732; C00157	0.0377
**KEGG pathways between YNX28H_vs_NJ5718**				
Linoleic acid metabolism	map00591	28/4834	C00157	0.1355
Pentose phosphate pathway	map00030	35/4834	C00198	0.1665
Arginine biosynthesis	map00220	23/4834	C00014	0.1127
Glycerolipid metabolism	map00561	38/4834	C00422	0.1795
Glutathione metabolism	map00480	38/4834	C00051	0.1795
Glycine, serine and threonine metabolism	map00260	50/4834	C00014	0.2294
beta-Alanine metabolism	map00410	32/4834	C01013	0.1533
Propanoate metabolism	map00640	48/4834	C01013	0.2213
alpha-Linolenic acid metabolism	map00592	44/4834	C00157	0.2048
Cysteine and methionine metabolism	map00270	64/4834	C00051	0.284
Glyoxylate and dicarboxylate metabolism	map00630	62/4834	C00014	0.2764
Pentose and glucuronate interconversions	map00040	58/4834	C00532	0.2611
Alanine, aspartate and glutamate metabolism	map00250	28/4834	C00152; C00014	0.0089
Starch and sucrose metabolism	map00500	37/4834	C00760; C00089	0.0153

Amino sugar and nucleotide sugar metabolism	map00520	108/4834	C00329; C00203	0.1064
Glycerophospholipid metabolism	map00564	56/4834	C00670; C00157; C00350	0.0028
**KEGG pathways between HY5H_vs_YNX28H**				
Pantothenate and CoA biosynthesis	map00770	30/4834	C00049	0.2309
Lysine biosynthesis	map00300	35/4834	C00049	0.264
Pentose phosphate pathway	map00030	35/4834	C00198	0.264
beta-Alanine metabolism	map00410	32/4834	C00049	0.2443
Fructose and mannose metabolism	map00051	54/4834	C00095	0.3774
Phenylpropanoid biosynthesis	map00940	68/4834	C01772; C05838; C01197	0.0207
Histidine metabolism	map00340	47/4834	C00025; C00049	0.0623
Galactose metabolism	map00052	46/4834	C00095; C00089	0.06
Pentose and glucuronate interconversions	map00040	58/4834	C00532; C00474	0.0898
Linoleic acid metabolism	map00591	28/4834	C00157	0.2173
Arginine biosynthesis	map00220	23/4834	C00025; C00049; C00014	0.001
Glycine, serine and threonine metabolism	map00260	50/4834	C00065; C00049; C00014	0.009
Glyoxylate and dicarboxylate metabolism	map00630	62/4834	C00025; C00065; C00014	0.0162
Arginine and proline metabolism	map00330	78/4834	C00025; C00148; C10497	0.0296
Glycerolipid metabolism	map00561	38/4834	C00416; C00422	0.0425
alpha-Linolenic acid metabolism	map00592	44/4834	C01226; C00157	0.0554

Amino sugar and nucleotide sugar metabolism	map00520	108/4834	C02336; C00329; C00203	0.0664
Alanine, aspartate and glutamate metabolism	map00250	28/4834	C00025; C00049; C00152; C00014	0.0001
Starch and sucrose metabolism	map00500	37/4834	C06215; C00208; C00089; C00095	0.0003
Cysteine and methionine metabolism	map00270	64/4834	C00049; C00170; C00065; C00051	0.0021
Glycerophospholipid metabolism	map00564	56/4834	C04230; C00416; C00065; C00157	0.0013
