# Supplementary material for: Rice Quality-Related Metabolites and the Regulatory Roles of Key Metabolites in Metabolic Pathways of High-Quality Semi-Glutinous japonica Rice Varieties
Source: Foods. 2022 Nov 17;11(22):3676. doi: 10.3390/foods11223676 (PMC9689214; doi:10.3390/foods11223676)
Supplement: Supplementary file 1 [file foods-11-03676-s001.zip › Table S6.pdf]

Table S6. Metabolite information related to rice quality traits and metabolic pathways.

Metabolite	Metab ID	KEGG Compound ID	HMDB Superclass	HMDB Class	HMDB Subclass
Sucrose	metab_7	C00089	Organic oxygen compounds	Organooxygen compounds	Carbohydrates and carbohydrate conjugates
Pantothenic Acid	metab_46	C00864	Organic acids and derivatives	Carboxylic acids and derivatives	Amino acids, peptides, and analogues
Sagittariol	metab_94	-	Lipids and lipid-like molecules	Prenol lipids	Diterpenoids
Glycerophosphocholine	metab_721	C00670	Lipids and lipid-like molecules	Glycerophospholipids	Glycerophosphocholines
4-Hydroxybenzaldehyde	metab_930	C00633	Organic oxygen compounds	Organooxygen compounds	Carbonyl compounds
Gamma-Eudesmol rhamnoside	metab_1395	-	Lipids and lipid-like molecules	Prenol lipids	Sesquiterpenoids
Oleoyl Ethanolamide	metab_1441	-	Organic nitrogen compounds	Organonitrogen compounds	Amines
L-Serine	metab_3953	C00065	Organic acids and derivatives	Carboxylic acids and derivatives	Amino acids, peptides, and analogues
L-Proline	metab_4020	C00148	Organic acids and derivatives	Carboxylic acids and derivatives	Amino acids, peptides, and analogues
2-Hydroxycinnamic acid	metab_4110	C01772;C05838	Phenylpropanoids and polyketides	Cinnamic acids and derivatives	Hydroxycinnamic acids and derivatives
Levan	metab_4116	C06215	Organic oxygen compounds	Organooxygen compounds	Carbohydrates and carbohydrate conjugates
Hydroxypropionic acid	metab_4160	C01013	Organic acids and derivatives	Hydroxy acids and derivatives	Beta hydroxy acids and derivatives
Goshonoside F1	metab_5663	-	Lipids and lipid-like molecules	Prenol lipids	Terpene glycosides
Euglobal IVa	metab_5965	-	Organic acids and derivatives	Carboxylic acids and derivatives	Carboxylic acid derivatives
Diosbulbinoside F	metab_6034	-	Lipids and lipid-like molecules	Prenol lipids	Terpene glycosides
Corchorifatty acid F	metab_7263	-	Lipids and lipid-like molecules	Fatty Acyls	Lineolic acids and derivatives
D-Maltose	metab_7885	C00208	Organic oxygen compounds	Organooxygen compounds	Carbohydrates and carbohydrate conjugates
L-Aspartic acid	metab_8037	C00049	Organic acids and derivatives	Carboxylic acids and derivatives	Amino acids, peptides, and analogues
Beta-Sitostenone	metab_2763	C00014	Homogeneous non-metal	Homogeneous other non-metal	_

			compounds	compounds	
L-Glutamate	metab_8388	C00025	Organic acids and derivatives	Carboxylic acids and derivatives	Amino acids, peptides, and analogues
L-Asparagine	metab_8054	C00152	Organic acids and derivatives	Carboxylic acids and derivatives	Amino acids, peptides, and analogues
Glutathione	metab_8387	C00051	Organic acids and derivatives	Carboxylic acids and derivatives	Amino acids, peptides, and analogues
Amylose	metab_8383	C00718	Organic oxygen compounds	Organooxygen compounds	Carbohydrates and carbohydrate conjugates
